# Characteristics of low affinity high capacity histamine uptake into neonatal rat astrocytes

**DOI:** 10.1186/2050-6511-13-S1-A19

**Published:** 2012-09-17

**Authors:** Mojca Kržan, Maša Novak, Sergej Pirkmajer, Katja Perdan-Pirkmajer

**Affiliations:** 1 Department of Pharmacology and Experimental Toxicology Faculty of Medicine, University of Ljubljana, 1000 Ljubljana, Slovenia; 2 Institute of Pathophysiology, Faculty of Medicine, University of Ljubljana, 1000 Ljubljana, Slovenia

## Background

The neurotransmitter histamine is synthesized from histidine in histaminergic neurons. Later on it is taken up into synaptic vesicles by the vesicular monoamine transporter 2 and released into the synaptic cleft upon depolarization stimuli. The released neurotransmitter is metabolised by the enzyme histamine N-methyltransferase (HNMT) producing tele-methylhistamine (tMH). In order to be enzymatically degraded or possibly recycled, histamine must be transported either into the presynaptic neuron or into surrounding glial cells. Unlike other neurotransmitters, the mechanism and the transporters by which the histamine content within the brain is regulated is currently unresolved.

## Methods

We used primary cultures of neonatal rat astrocytes to determine kinetic properties of histamine uptake and HNMT and organic cation transporter (OCT) mRNA expression. In addition, we investigated the influence of different antidepressants and OCT inhibitors on histamine transport into astrocytes

## Results

Specific uptake of [^3^H]histamine increased in a time-, temperature- and Na^+^-dependent and ouabain-sensitive manner. The Na^+^-dependent [^3^H]histamine uptake was saturable. The K_m_ value for this process was around 100 M and V_max_ was 160 pmol/mg protein/min, resembling low-affinity high-capacity uptake 2, which might occur via OCT2, the OCT isoform expressed in astrocytes. [^3^H]histamine uptake was inhibited only by amitriptyline and desipramine, whereas the histamine metabolite tMH affected both histamine transport and reverse transport from cultured astrocytes. On the other hand, neither decynium-22 nor corticosterone, known inhibitors of OCT, affected carrier-operated histamine transport.

## Conclusions

Taken together, astrocytes can represent a major inactivation site for histamine, but some facts remain unresolved, such as the existence of specific histamine transporters, the involvement of non-selective transporters and a possible release of histamine and/or its metabolites from astrocytes.

